# A Novel Peptide Oligomer of Bacitracin Induces M1 Macrophage Polarization by Facilitating Ca^2+^ Influx

**DOI:** 10.3390/nu12061603

**Published:** 2020-05-29

**Authors:** Seon Yeong Ji, Hyesook Lee, Hyun Hwangbo, Su-Hyun Hong, Hee-Jae Cha, Cheol Park, Do-Hyung Kim, Gi-Young Kim, Suhkmann Kim, Heui-Soo Kim, Jin Cheol Yoo, Yung Hyun Choi

**Affiliations:** 1 Anti-Aging Research Center, Dong-eui University, Busan 47340, Republic of Korea; 14602@deu.ac.kr (S.Y.J.); lhyes0219@pusan.ac.kr (H.L.); hbhyun2003@naver.com (H.H.); hongsh@deu.ac.kr (S.-H.H.); 2 Department of Biochemistry, College of Korean Medicine, Dong-eui University, Busan 47227, Republic of Korea; 3 Department of Parasitology and Genetics, College of Medicine, Kosin University, Busan 49104, Republic of Korea; hcha@kosin.ac.kr; 4 Department of Molecular Biology, College of Natural Sciences, Dong-eui University, Busan 47340, Republic of Korea; parkch@deu.ac.kr; 5 Department of Aquatic Life Medicine, College of Fisheries Science, Pukyong National University, Busan 48513, Republic of Korea; dhkim@pknu.ac.kr; 6 Department of Marine Life Sciences, School of Marine Biomedical Sciences, Jeju National University, Jeju 63243, Republic of Korea; immunkim@jejunu.ac.kr; 7 Department of Chemistry, College of Natural Sciences, Pusan National University, Busan 46241, Republic of Korea; suhkmann@pusan.ac.kr; 8 Department of Biological Sciences, College of Natural Sciences, Pusan National University, Busan 46241, Republic of Korea; khs307@pusan.ac.kr; 9 Department of Pharmacy, College of Pharmacy, Chosun University, Gwangju 61452, Republic of Korea; jcyu@chosun.ac.kr

**Keywords:** antimicrobial peptides, CSP32, immune, macrophage polarization, inflammation, Ca^2+^

## Abstract

Antimicrobial peptides (AMPs) are components of the innate immune system and form the first defense against pathogens for various organisms. In the present study, we assessed whether CSP32, a novel AMP oligomer of bacitracin isolated from a strain of *Bacillus* spp., regulates the polarization of murine macrophage-like RAW 264.7 cells. CSP32 stimulated phagocytosis while inducing the appearance of the typical M1 polarized macrophage phenotype; these M1 macrophages play a role in host defense against pathogens. Furthermore, our results showed that CSP32 enhanced the expression and production of pro-inflammatory mediators, such as cytokines and chemokines. In addition, the CSP32-stimulated inflammatory mediators were induced mainly by the mitogen-activated protein kinase/nuclear factor kappa B (MAPK/NF-κB) signaling pathway during M1 macrophage polarization. In particular, CSP32 markedly increased the numbers of Ca^2+^-positive macrophages while upregulating phospholipase C and activating protein kinase Cε. Furthermore, the inhibition of intracellular Ca^2+^ by BAPTA-AM, a Ca^2+^ chelator, significantly suppressed the CSP32-mediated phagocytosis, inflammatory mediator production, and NF-κB activation. In conclusion, our data suggested that CSP32-stimulated M1 macrophage polarization is dependent on the calcium signaling pathway and may result in enhanced immune capacities.

## 1. Introduction

Macrophages are phagocytic cells of the immune system that are located in various tissues [1]. In innate and adaptive immunity, macrophages are responsible for defending against pathogens and are involved in the recognition, processing, and presentation of antigens to T cells [2,3]. Macrophage polarization (MP) is a process by which different phenotypes, functions, and transcriptional profiles are obtained in response to surrounding microenvironmental signals; macrophages are commonly polarized into two distinct subsets, namely, M1 and M2 [4,5]. Classically activated M1 macrophages play a role in host defense against pathogens and are able to inhibit immunosuppressive cells and promote T helper type 1 (Th1) responses through the production of pro-inflammatory and Th1 cytokines [3,6]. By contrast, alternatively activated M2 macrophages are involved in the anti-inflammatory response through the secretion of Th2 cytokines and anti-inflammatory cytokines [5]. It is known that the balance of M1/M2 polarization governs the fate of the immune system, injury and inflammation in organs [7]. In this regard, macrophages initially exhibit the M1 phenotype in response to infection, and the stimulation of the immune system via the acquisition of the M1 polarized macrophage phenotype is one of the key strategies to enhance the human defense system.

Antimicrobial peptides (AMPs) exist in various organisms and act as host defense peptides that have potent antimicrobial activity against bacteria, fungi, and viruses [8,9]. AMPs are components of innate immunity, and these peptides can bind to the cell membrane of pathogens, leading to the destruction of the cell membrane, permeation of proteins, and destruction of cell morphology, eventually resulting in cell death [8]. *Bacillus* has been found in almost all environments, including plants, animals, soil, and seawater [10]. *Bacillus* has various phenotypes, including Gram-positive or Gram-variable, aerobic or facultative anaerobic, and rod-shaped or endospore-forming bacteria [11]. *Bacillus* spp. are a rich source of AMPs that served as an efficient source of antibiotic. [12,13]. For these reasons, *Bacillus* spp. are widely used in numerous biotechnological fields, including the pharmaceutical and food industries [12,14]. Recently, Choi et al. showed that CSP32 was purified from a strain of *Bacillus* spp., which was isolated from traditional Korean fermented foods, and that CSP32 is a novel oligomer of bacitracin [15]. CSP32 has a molecular mass of 5697.9 Da and the first 12 amino acids of the N terminus of CSP32 were found to be APLEXXIFHDN. The sequence has a high degree of similarity to bacitracin, which is an antibiotic produced by certain species [15]. Furthermore, we have demonstrated that CSP32 has antimicrobial activity against methicillin-resistant *Staphylococcus aureus*, vancomycin-resistant *S. aureus*, vancomycin-resistant enterococci, *Propionibacterium acne* and *Clostridium difficile*, and anti-inflammatory properties in lipopolysaccharide (LPS)-induced inflammation in RAW 264.7 macrophage cells [15]. Based on these results, the aim of the current study was to investigate the effect of CSP32, a novel peptide oligomer of bacitracin from a strain of *Bacillus* spp., on immune responses. To evaluate the effect of CSP32 on immunity, we assessed whether CSP32 regulates the polarization of murine macrophage-like RAW 264.7 cells.

## 2. Materials and Methods

### 2.1. Chemicals and Reagents

Lipopolysaccharide (LPS; Escherichia coli Serotype, 055: B5), sulfanilamide, N-(1-Naphthyl) ethylenediamine dihydrochloride (NED), BAPTA-AM, 4′,6-diamidino-2-phenylindole (DAPI), U73122 and phosphoric acid were purchased from Sigma-Aldrich Chemical Co. (St. Louis, MO, USA). Fluo-3-AM and NE-PER Nuclear/Cytoplasmic Extraction Reagents were obtained from Thermo Fisher Scientific Inc. (Waltham, MA, USA). 3-(4,5-Dimethylthiazol-2-yl)-2,5-diphenyltetrazolium bromide (MTT) and 5,6-carboxy-2′,7′-dichlorodihydrofluorescein diacetate (DCF-DA) were obtained from Invitrogen (Carlsbad, CA, USA). Interleukin (IL)-1β (catalog No. MLB00C), monocyte chemoattractant protein (MCP)-1 (catalog no. MJE00) and tumor necrosis factor (TNF)-α (catalog no. SMTA00B) enzyme-linked immunosorbent assay (ELISA) kits were purchased from R&D Systems, Inc. (Minneapolis, MN, USA). A prostaglandin E_2_ (PGE_2_) ELISA kit (catalog no. 500141) and phagocytosis assay kit (catalog no. 500290) were obtained from Cayman Chemical (Ann Arbor, MI, USA). A nuclear factor kappa B (NF-κB) p65 transcription factor assay kit (catalog no. ab133112) was purchased from Abcam Inc. (Cambridge, UK).

### 2.2. Preparation of CSP32

CSP32 was isolated and purified from newly isolated *Bacillus* spp. CS32 as previously described [15]. Prior to use in the experiments, CSP32 was dissolved in distilled water and diluted to the required concentrations in culture medium just before use.

### 2.3. Cell Culture and Viability Analysis

Murine macrophage-like RAW 264.7 cells were obtained from the Korea Cell Line Bank (Seoul, Korea) and were cultured in Dulbecco’s modified Eagle’s medium (DMEM; WelGENE Inc., Daegu, Korea) supplemented with 10% fetal bovine serum (FBS, WelGENE Inc.). The RAW 264.7 cells were grown to 80–90% confluence and maintained in an incubator at 37 °C in an atmosphere of 5% CO_2_. The cell viability was assessed by MTT as previously described [16]. In brief, RAW 264.7 cells were seeded on 96-well plates at a density of 1 × 10^4^ cells/well and incubated for 24 h. The cells were treated with the desired concentrations of CSP32 (17.6 and 88.0 μM) and l ng/mL LPS. A super-low dose of LPS (<1 ng/mL) is the physiologically relevant concentration that was used as a positive control [17,18]. After 24 h, the cells were incubated with 50 μg/mL MTT solution for 2 h, dissolved in dimethylsulfoxide (Sigma-Aldrich Chemical Co.), and then analyzed at 540 nm by a microplate reader (VERSA Max, Molecular Device Co., Sunnyvale, CA, USA).

### 2.4. Nitic Oxide (NO) Assay

RAW 264.7 cells were seeded on 6-well plates at a density of 4 × 10^5^ cells/well, and incubated for 24 h. The cells were treated with CSP32 and l ng/mL LPS for 24 h, and then, the culture supernatants were harvested to assess the NO levels. The NO levels were measured by the Griess reaction, and the optical absorbance was detected at 540 nm using a microplate reader [19].

### 2.5. Phagocytosis Analysis

As described previously, the phagocytic activity was measured using a phagocytosis assay kit [20]. In brief, RAW 264.7 cells were seeded on 6-well plates at a density of 4 × 10^5^ cells/well, and incubated for 24 h. The cells were treated with CSP32 and l ng/mL LPS for 24 h, and then, a fluorescently labeled latex bead-rabbit IgG-FITC complex was added for 2 h. The cells were washed and treated with 40 μM DAPI. After incubation for 10 min, the fluorescence intensity was assessed by flow cytometry and fluorescence microscopy (Leica Microsystems, Wentzler, Germany).

### 2.6. Gene Expression Microarray Analysis

The nCounter SPRINT platform (NanoString Technologies, Inc. Seattle, WA, USA) was used to analyze the gene expression by microarray, as described previously [21]. Briefly, RAW 264.7 cells were seeded on 6-well plates at a density of 4 × 10^5^ cells/well, and incubated for 24 h. The cells were treated with CSP32 and l ng/mL LPS for 24 h, and then, the total RNA was isolated by TRIzol reagent (Invitrogen) according to the manufacturer’s instructions. To evaluate for RNA condition, all samples were performed quality control test and quantitative analysis using AATI fragment analyzer (Agilent Technologies, Santa Clara, CA, USA) and DS-11 spectrophotometer (DeNovix Inc., Wilmington, DE, USA). After solution-phase hybridization between the target mRNA and reporter-capture probe pairs, the excess probe was removed, and the probe/target complexes were aligned and immobilized in the nCounter cartridge (NCT-120), which was then placed in a digital analyzer for image acquisition and data processing. The raw data were normalized with 14 housekeeping genes, including ALAS1, EEF1G, G6PDX, GAPDH, GUSB, HPRT, OAZ1, POLR1B, POLR2A, PPIA, RPI19, SDHA, TBP, and TUBB5. Fold change data was log2-transformed and log2 fold-change cut-offs of −2.5 and 2.5 (red and green, respectively) in the expression of the selected genes are presented in a heat map.

### 2.7. Levels of TNF-α, Interleukin (IL)-1β, MCP-1, and PGE_2_

RAW 264.7 cells were seeded on 6-well plates at a density of 4 × 10^5^ cells/well, and incubated for 24 h. The cells were treated with CSP32 and l ng/mL LPS for 24 h, and then, the culture supernatants were harvested to analyze the levels of TNF-α, IL-1β, MCP-1, and PGE_2_ according to the manufacturer’s instructions.

### 2.8. Reverse Transcriptase-Polymerase Chain Reaction (RT-PCR)

RAW 264.7 cells were seeded on 6-well plates at a density of 4 × 10^5^ cells/well, and incubated for 24 h. The cells were treated with CSP32 and l ng/mL LPS for 24 h, and then, the total RNA was isolated from the cells using TRIzol reagent (Invitrogen) according to the manufacturer’s instructions. cDNA was synthesized using the AccuPower^®^ PCR PreMix (Bioneer, Daejeon, Korea), as previously described [22]. The primer sequences used were as follows: cyclooxygenase-2 (COX-2; GenBank ID: NM-011198), sense (5′- GCGACATACTCAAGCAGGAGCA-3′) and antisense (5′- AGTGGTAACCGCTCAGGTGTTG-3′); glyceraldehyde 3-phosphate dehydrogenase (GAPDH, GenBank ID: NM-008084), sense (5′-CATCACTGCCACCCAGAAGACTG-3′) and antisense (5′-ATGCCAGTGAGCTTCCCGTTCAG-3′); IL-1β (GenBank ID: NM-008361), sense (5′-TGGACCTTCCAGGATGAGGACA-3′) and antisense (5′-GTTCATCTCGGAGCCTGTAGTG-3′); IL-6 (GenBank ID: NM-031168), sense (5′-TACCACTTCACAAGTCGGAGGC-3′) and antisense (5′- CTGCAAGTGCATCATCGTTGTTC-3′); inducible nitric oxide synthase (iNOS, GenBank ID: NM-010927), sense (5′- GAGACAGGGAAGTCTGAAGCAC-3′), and antisense (5′- CCAGCAGTAGTTGCTCCTCTTC-3′); and TNF-α (GenBank ID: NM-013693), sense (5′-GGTGCCTATGTCTCAGCCTCTT-3′) and antisense (5′-GCCATAGAACTGATGAGAGGGAG-3′). The PCR products were separated by 1% agarose gel electrophoresis and stained with ethidium bromide (Sigma-Aldrich Chemical Co.), and the gels were visualized with UV illumination. The immunoreactive bands were visualized by a Fusion FX Image system (Vilber Lourmat, Torcy, France). Densitometric analysis of the data was performed using the ImageJ^®^ software (v1.48, NIH, Bethesda, MD, USA).

### 2.9. Western Blot Analysis

RAW 264.7 cells were seeded on 100 mm culture dish at a density of 2 × 10^6^ cells/well, and incubated for 24 h. The cells were treated with CSP32 and l ng/mL LPS for 24 h, and then, the total protein was extracted from the cells using a Bradford protein assay kit (Bio-Rad Laboratories, Hercules, CA, USA). A total of 40 μg of protein was separated by sodium-dodecyl sulfate-polyacrylamide gel electrophoresis and transferred to polyvinylidene difluoride membranes (Merck Millipore, Bedford, MA, USA). The membranes were blocked using 5% skim milk in Tris-buffered saline containing 0.1% Triton X-100 (TBST) at room temperature (RT) for 1 h and probed with specific primary antibodies at 4 °C overnight. Subsequently, the membranes were incubated with the corresponding secondary antibodies for 1 h at RT, developed, and analyzed using a Fusion FX Image system (Vilber Lourmat, Torcy, France). Densitometric analysis of the data was performed using the ImageJ^®^ software (v1.48, NIH, Bethesda, MD, USA). The information about the antibodies is provided in Table S1.

### 2.10. Intracellular Calcium Analysis

RAW 264.7 cells were seeded on 6-well plates at a density of 4 × 10^5^ cells/well, and incubated for 24 h. The cells were treated with CSP32 and l ng/mL LPS for 24 h. To investigate the CSP32- or LPS-mediated calcium trafficking, the cytosolic calcium was assessed by co-incubating the cells with 2 μM fluo-3-AM, a cell permeant Ca^2+^ indicator, for 30 min [23]. The changes in the cytoplasmic calcium were estimated by flow cytometry and fluorescence microscopy.

### 2.11. Analysis of NF-κB Activation

RAW 264.7 cells were seeded on a 100 mm culture dish at a density of 2 × 10^6^ cells/well, and incubated for 24 h. The cells were treated with CSP32 and l ng/mL LPS for 24 h. Nuclear-cytoplasmic fractionation was performed using the NE-PER Nuclear and Cytoplasmic Extraction Reagents kit (Thermo Fisher Scientific Inc., Rockford, IL, USA) according to the manufacturer’s protocol. The purities of the cellular fractions were determined by western blot analysis. Lamin B1 and actin served as the internal controls for the nuclear and cytoplasmic fractions, respectively. To assess the NF-κB activity, RAW 264.7 cells were seeded on 6-well plates at a density of 4 × 10^5^ cells/well, and incubated for 24 h. The cells were treated with CSP32 and l ng/mL LPS for 24 h, and then, the nuclear fraction was analyzed using the NF-κB p65 transcription factor assay kit according to the manufacturer’s instructions. Furthermore, NF-κB p65 nuclear localization was observed by immunofluorescence assays. In brief, the cells were seeded on 4-well chamber slide (SPL Life Sciences Co., Pocheon, Korea) at a density of 2 × 10^5^ cells/well, and incubated for 24 h. Subsequently, the cells were treated with CSP32 or LPS and then incubated with anti-NF-kB antibody at 4 °C overnight. The cells were probed with Alexa Fluor 488-labeled donkey anti-rabbit IgG (Thermo Fisher Scientific, Waltham, MA, USA) antibody for 1 h in the dark. The position of the cell nucleus was assessed with DAPI. The cells were mounted and images were captured using a fluorescence microscope.

### 2.12. Statistical Analysis

All the experiments were performed by conducting each assay at least three times. The data were analyzed using GraphPad Prism 5.03 (GraphPad Software Inc., La Jolla, CA, USA) and are expressed as the mean ± standard deviation (SD). The statistical analyses were conducted using analysis of variance (ANOVA) and Tukey’s test to examine between-group differences, and *p* < 0.05 was considered significant.

## 3. Results

### 3.1. CSP32 Induced Morphological Changes and Phagocytosis of Macrophages

**Figure 1:**
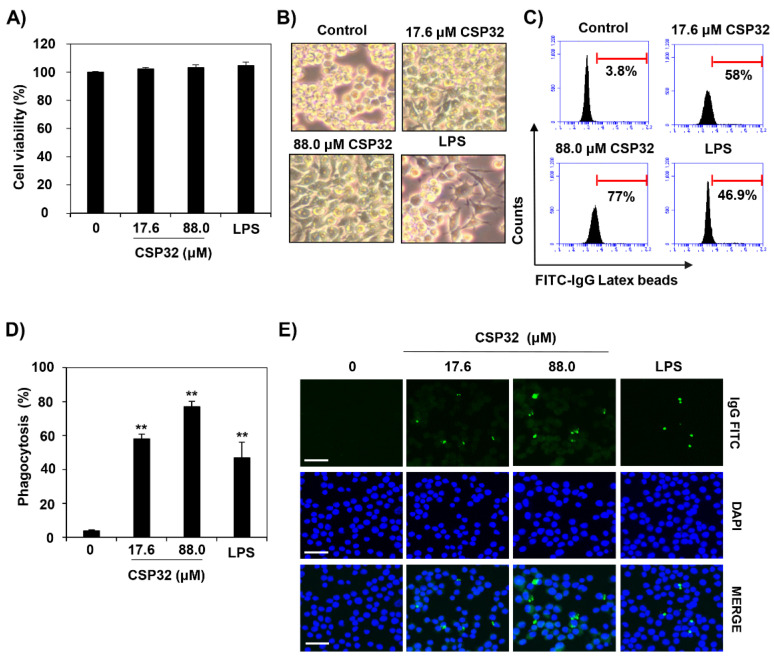
CSP32 induced morphological changes and phagocytosis of macrophages. Cells were treated with the indicated concentrations of CSP32 and LPS for 24 h. (**A**) Cell viability was assessed by MTT assay. (**B**) Representative microscopy images of morphological changes. (**C**,**D**) Representative flow cytometric histogram and quantitative analysis of the phagocytosis capacity using fluorescent FITC-IgG latex beads. Data are expressed as the mean ± SD (*n* = 4). ** *p* <0.01 compared with the control. (**E**) The phagocytic cells were visualized by fluorescence microscopy. The nuclei were stained with DAPI. Scale bar: 200 μm.

To estimate the effect of CSP32 on cell viability, cells were incubated with CSP32 for 24 h and then treated with MTT solution for 2 h. Up to 88.0 μM of CSP32 exerted no cytotoxicity in RAW 264.7 cells (Figure 1A). As shown in Figure 1B, CSP32-treated cells appeared spindle-shaped with elongated filopodia, whereas untreated cells exhibited a round form. Meanwhile, 1 ng/mL LPS, a positive control, did not affect cell viability and caused the cells to adopt an irregular shape. To investigate the effect of CSP32 on phagocytosis, the cells were treated with CSP32 or LPS for 24 h, after which the intensity of the fluorescently labeled FITC-conjugated latex bead complex was assessed using flow cytometry and fluorescence microscopy. Immunological phagocytic recognition by monocytes and macrophages can be mediated by cell surface receptor for IgG, IgM, and a modified component of complement [24]. The quantification of phagocytic cells via flow cytometry indicated that macrophages treated with 17.6 μM and 88.0 μM CSP32 exhibited markedly elevated phagocytosis by approximately 15-fold and 20-fold compared with the control, respectively (Figure 1C,D). In addition, when the cells were incubated with CSP32, the macrophages contained a substantially higher percentage of FITC-IgG-positive phagocytosing cells (Figure 1E). LPS also enhanced the phagocytosis activity by up to 14-fold compared with the control treatment, and the levels were similar to those of the 17.6 μM CSP32 treatment.

### 3.2. CSP32 Upregulated the Expression of Macrophage Polarization-Related Genes

**Figure 2:**
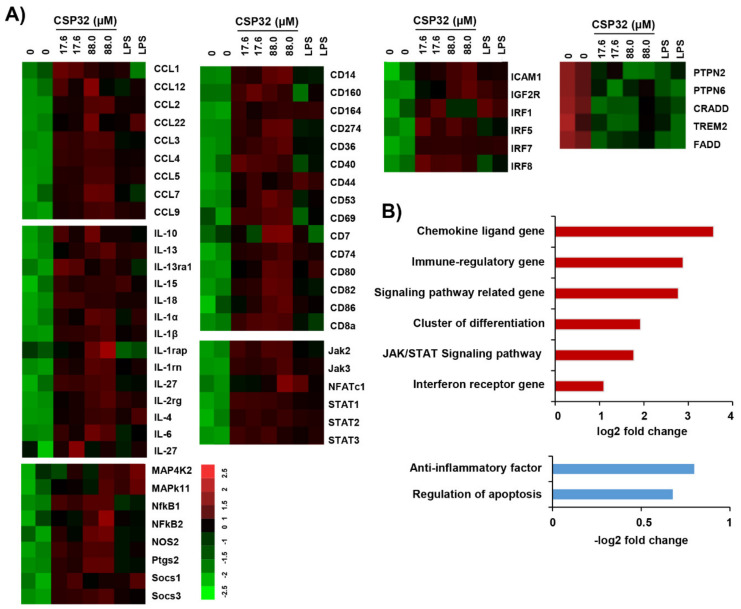
Microarray gene expression analysis of CSP32 in macrophages. Total RNA was collected by harvesting the CSP32- and LPS-treated cells after 24 h (*n* = 2). (**A**) Microarray heatmap representing the log2 fold-change of macrophage polarization-regulated gene expression. Data are expressed as a cut-off of −2.5 to 2.5 (red to green, respectively). (**B**) Histogram shows the upregulated genes (upper panel) and downregulated genes (bottom panel) ranked by log2 fold-change in expression between untreated cells and 88.0 μM CSP32-treated cells.

We assessed the profile of macrophage polarization-related gene expression using the nCounter SPRINT platform. Figure 2A shows the heatmap of the selected genes and fold changes in the expression of these genes in the treated cells compared with the untreated cells. CSP32 upregulated the expression of interleukins, CC chemokine ligands (CCLs), cluster of differentiation and immune regulatory genes, and the levels of these genes were enhanced by over 1.5-fold compared with those in the control cells. Clearly, the expression levels of the CCL genes, including CCL1~9, CCL12, and CCL 22, were markedly increased by 3.72-log2-fold in the CSP32-treated cells (Figure 2B). In addition, 88.0 μM CSP32 increased the expression of the cluster of differentiation and immune regulatory genes by 1.99-log2-fold and 3.08-log2-fold, respectively (Figure 2B). Specifically, the expression levels of markers of M1 macrophages, such as iNOS and PGE_2_, were remarkably increased by CSP32 treatment (Figure 2A). Moreover, the M1 phenotype-related NF-κB signaling pathway was substantially upregulated in the CSP32-treated cells. In addition, the levels of genes related to the Janus kinase/signal transducer and activator of transcription (JAK/STAT) signaling pathway were slightly upregulated by 1.74-log2-fold due to CSP32 treatment. Moreover, LPS also increased the expression of inflammatory mediators, including cytokines and chemokines. Meanwhile, CSP32-treated cells downregulated the expression of anti-inflammatory factors, including protein tyrosine phosphatase non-receptor type 2 (PTPN2), PTPN6, and triggering receptor expressed on myeloid cells 2 (TREM2). Furthermore, the expression of apoptosis-regulatory genes also suppressed by CSP32 (Figure 2B). A basis for the histogram of Figure 2B provided in Figure S1.

### 3.3. CSP32 Increased the Levels of Markers of M1 Macrophages

**Figure 3:**
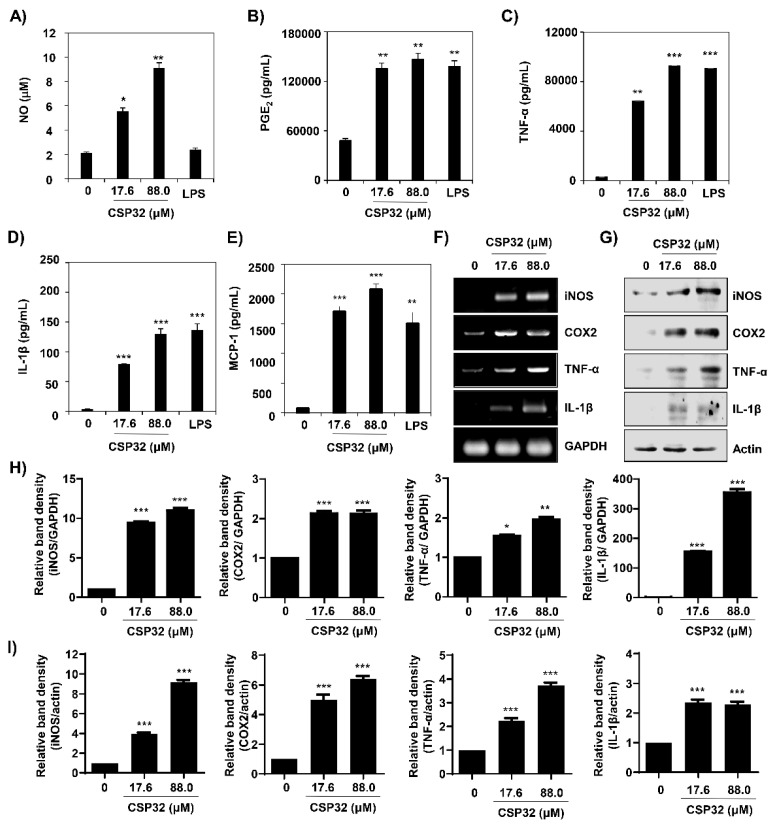
CSP32 increased the levels of markers for M1 macrophages. Cells were treated with the indicated concentrations of CSP32 and LPS for 24 h. (**A**) The amount of NO in the cell supernatant was measured using Griess reagents. The levels of PGE_2_ (**B**), TNF-α (**C**), IL-1β (**D**), and MCP-1 (**E**) in the culture supernatants were measured by ELISA kits. Data are expressed as the mean ± SD (*n* = 4). * *p* < 0.05, ** *p* < 0.01, and *** *p* < 0.001 compared with the control. mRNA (**F**) and protein (**G**) expression of markers of M1 macrophages, including iNOS, COX-2, TNF-α, and IL-1β. GAPDH and β-actin were used as internal controls for RT-PCR and Western blotting. Quantitative analysis of mRNA (**H**) and protein (**I**) expression. The expression of each protein was indicated as a fold change relative to the control. Data are expressed as the mean ± SD (*n* = 3). * *p* < 0.05, ** *p* < 0.01, and *** *p* < 0.001 compared with the control.

Based on the microarray gene expression results, we subsequently validated whether CSP32 upregulated the secretion and expression of M1 macrophage markers. Figure 3A shows that the amount of NO was significantly increased by CSP32 in a dose-dependent manner (17.6 μM CSP32: 5.73 ± 0.25, 2.86-fold of control, *p* < 0.05; 88.0 μM CSP32: 9.18 ± 0.34, 4.59-fold of control, *p* < 0.01) but was not changed by LPS. Furthermore, the ELISA results showed that the levels of markers of M1 macrophages—including PGE_2_, TNF-α, IL-1β, and MCP-1—were remarkably enhanced by CSP32 and LPS (Figure 3B–E). To further validate the expression of representative markers of M1 macrophages, RT-PCR and western blot analysis were employed to assess the expression of iNOS, COX-2, TNF-α, and IL-1β. Our results showed that the mRNA and protein expression levels of M1 markers were markedly increased by CSP32 treatment, and this finding is consistent with the ELISA results (Figure 3F–I).

### 3.4. CSP32 Activated the MAPKs and NF-κB Signaling Pathways in Macrophages

**Figure 4:**
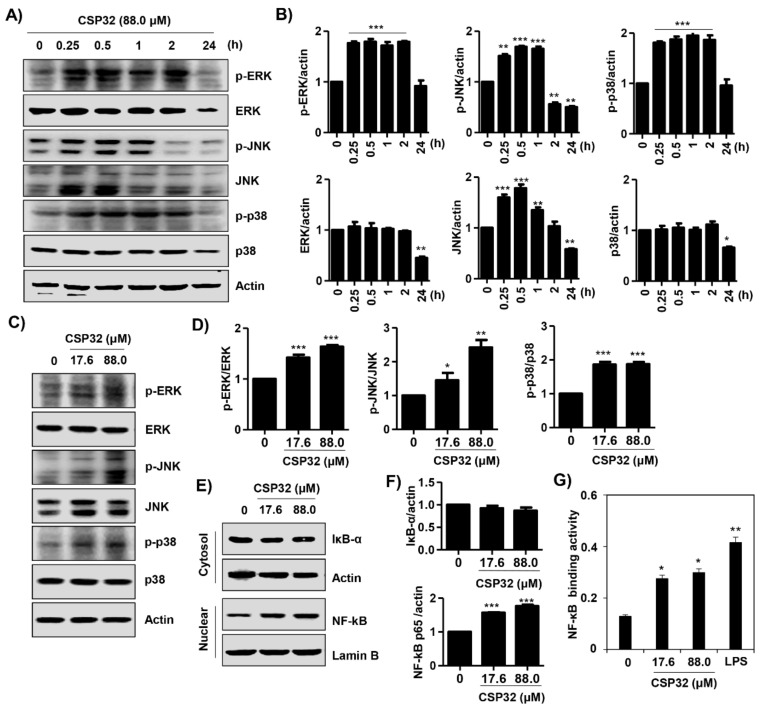
CSP32 activated MAPKs and the NF-κB signaling pathway in macrophages. (**A**) At 0, 15 min, 30 min, 1 h, 2 h, and 24 h after CSP32 treatment, cells were harvested and lysed. (**C**,**E**) Cells were treated with the indicated concentration of CSP32 for 1 h and subsequently harvested and lysed. (**A**,**C**) Total cell lysates were examined by western blotting for ERK, JNK, and p38 MAPK phosphorylation. β-actin was used as an internal control. (**E**) Cytoplasmic and nuclear lysates were examined by western blotting for IκB-α and NF-κB. Actin and lamin B1 serve as the internal controls for the cytoplasmic and nuclear lysates, respectively. (**B**,**D**,**F**) Quantitative analysis of protein expression. The expression of each protein was indicated as a fold change relative to the control. Data are expressed as the mean ± SD (*n* = 3). * *p* < 0.05, ** *p* < 0.01 and *** *p* < 0.001 compared with the control. (**G**) In nuclear lysates, NF-κB activity was analyzed using an NF-κB p65 transcription factor assay kit. Data are expressed as the mean ± SD (*n* = 4). * *p* < 0.05 and ** *p* < 0.01 compared with the control.

Following the microarray analyses, we verified that the expression of genes related to the JAK, STAT, MAPKs, and NF-κB signaling pathways was upregulated in CSP32-treated cells. Based on this result, we next confirmed the effect of CSP32 on the expression of proteins related to the JAK/STAT and MAPK/NF-κB signaling pathways according to time point and concentration. The phosphorylation of extracellular signal-regulated kinase (ERK) and p38 MAPK was markedly increased at 2 h following 88.0 μM CSP32 treatment, while the phosphorylation of c-Jun N-terminal kinases (JNK) was highest at 1 h (Figure 4A,B). In addition, we verified that both 17.6 μM and 88.0 μM CSP32 also increased the phosphorylation of ERK, JNK, and p38 after 1 h of treatment (Figure 4C,D). In Figure 4E,F, we showed that CSP32 simultaneously upregulated the expression of nuclear NF-κB p65. This CSP32-mediated NF-κB p65 activation was reconfirmed by analysis of NF-κB p65 binding activity. Our data showed that CSP32 significantly increased the NF-κB p65 binding activity in the nucleus (Figure 4G). Moreover, LPS also enhanced the NF-κB p65 binding activity in the nucleus.

### 3.5. CSP32 Stimulated Cytosolic Ca^2+^ Influx During M1 Polarization

**Figure 5:**
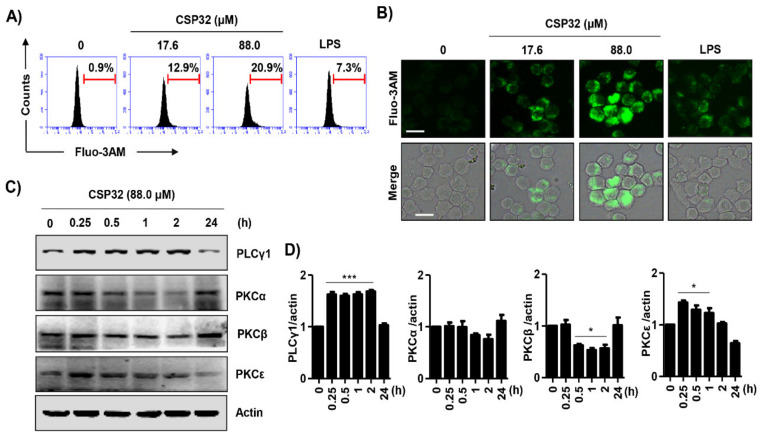
CSP32 upregulated cytosolic Ca^2+^ concentrations during M1 polarization. Cells were treated with the indicated concentrations of CSP32 and LPS for 24 h. Then, cells were stained with 2 μM fluo-3-AM for 30 min. (**A**) Representative histogram from the flow cytometric analysis of fluo-3A-M-positive macrophages following CSP32 and LPS treatment. (**B**) Representative fluorescence images. Scale bar; 20 μm. (**C**) At 0, 15 min, 30 min, 1 h, 2 h and 24 h after CSP32 treatment, cells were harvested and lysed. Total cell lysates were examined by western blotting for PLCγ1, PKCα, PKCβ, and PKCε. β-actin was used as an internal control. (**D**) Quantitative analysis of protein expression. The expression of each protein was indicated as a fold change relative to the control. Data are expressed as the mean ± SD (*n* = 3). * *p* < 0.05 and *** *p* < 0.001 compared with the control.

We examined the effect of CSP32 on Ca^2+^ concentrations, as indicated by fluo-3-AM fluorescence intensity and analyzed by flow cytometry. As shown in Figure 5A, CSP32 increased the cytosolic Ca^2+^ concentration in a dose-dependent manner. Moreover, to reconfirm the influence of CSP32 on the changes in calcium concentrations, we observed calcium concentrations under fluorescence microscopy. Consistent with the flow cytometry results, CSP32 increased the number of fluo-3-AM-positive RAW264.7 cells, whereas LPS slightly upregulated the fluorescence intensity (Figure 5B). We next investigated whether the phospholipase C (PLC)/protein kinase C (PKC) signaling pathway is involved in the CSP32-mediated intracellular Ca^2+^ influx. In the present study, we found that the expression of PLCγ1 was upregulated to approximately 1.68-fold of control by 88.0 μM CSP32 within 2 h (Figure 5C,D). Furthermore, CSP32 gradually upregulated the expression of PKCε but not classical PKCs, including PKCα and PKCβ. However, these alterations in the expression of PLCγ1 and PKCε were completely downregulated at 24 h. Additionally, to evaluate whether the inhibition of PLC impact the signaling pathways triggered by CSP32, we have performed additional test using U73122, a PKC inhibitor. As a result, we found that pre-treatment of 10 μM U73122 markedly blocked the upregulation of CPS32-mediated PLC and PKCε. In addition, we found that U73122 significantly downregulated the expression of CSP32-induced MAPK/NF-κB signaling pathway (Figure S2). Based on this result, we confirmed that CSP32-stimulated M1 polarization is dependent on the PLCγ signaling pathway.

### 3.6. CSP32-Mediated Ca^2+^ Influx Regulated M1 Polarization

**Figure 6:**
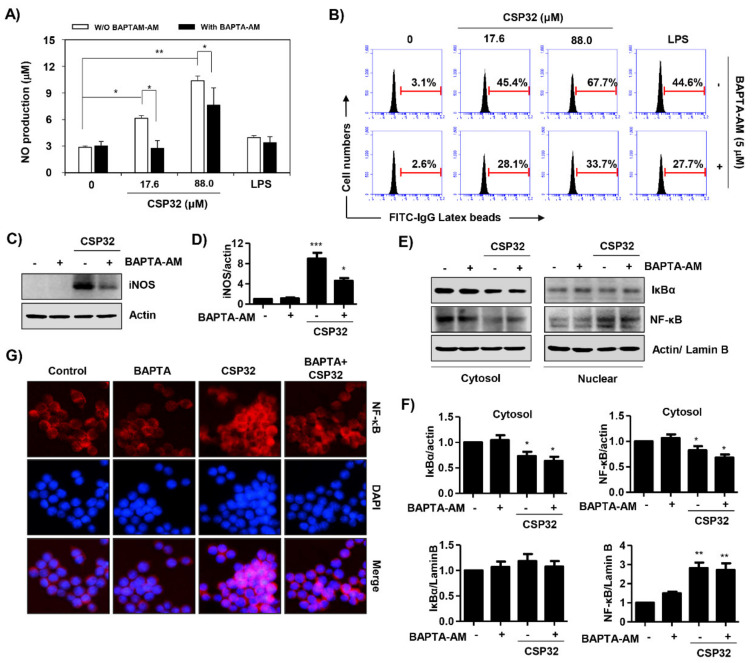
Ca^2+^ regulated CSP32-mediated M1 polarization. (**A**–**C**) Cells were pretreated with or without 5 μM BAPTA-AM for 30 min and then incubated with the indicated concentrations of CSP32 and LPS for 24 h. (**A**) The amount of NO in the cell supernatants was measured using Griess reagents. Data are expressed as the mean ± SD (*n* = 4). * *p* < 0.05 and ** *p* < 0.01 compared between the groups. (**B**) Representative flow cytometric histogram of the phagocytosis capacity using fluorescent FITC-IgG latex beads. (**C**) Protein expression of iNOS. β-actin was used as an internal control for total cell lysates. (**D**) Quantitative analysis of iNOS expression that was indicated as a fold change relative to the control. Data are expressed as the mean ± SD (*n* = 3). * *p* < 0.05 and *** *p* < 0.001 compared with the control. (**E**,**F**) Cells were pretreated with or without 5 μM BAPTA-AM for 30 min and then incubated with the indicated concentration of 5 μg/mL CSP32 for 1 h. (**E**) Cytoplasmic and nuclear lysates were examined by western blotting for IκB-α and NF-κB. Actin and lamin B1 serve as the internal controls for the cytoplasmic and nuclear lysates, respectively. (**F**) Quantitative analysis of IκBα and NF-κB p65 expression that was indicated as a fold change relative to the control. Data are expressed as the mean ± SD (*n* = 3). * *p* < 0.05 and ** *p* < 0.01 compared with the control. (**G**) Localization of NF-κB p65 (green) was visualized with fluorescence microscopy. Cells were stained with DAPI for visualization of the nuclei (blue).

Based on the results showing that CSP32 induced the cytosolic Ca^2+^ influx, we verified whether Ca^2+^ directly regulates CSP32-induced M1 polarization. Our findings showed that pretreatment with BAPTA-AM, a Ca^2+^ chelator, significantly suppressed the CSP32-induced NO production (Figure 6A) and iNOS expression (Figure 6C,D). In addition, the upregulation of phagocytosis activity by CSP32 treatment was also markedly suppressed in the BAPTA-AM-coincubated cells (Figure 6B). BAPTA-AM treatment also markedly suppressed the LPS-induced phagocytosis activity. Furthermore, we found that pretreatment with BAPTA-AM reversed the upregulation of nuclear NF-κB p65 and the downregulation of cytosolic NF-κB p65 following CSP32 (Figure 6E,F). These results coincide with the results of the immunofluorescence assay (Figure 6G).

## 4. Discussion

Macrophages are effector cells of the innate immune system that phagocytose pathogens and then coordinate the adaptive immune response [25,26]. Phagocytosis by macrophages, which is a representative feature of activated macrophages, plays a critical role in the uptake and degradation of pathogens and senescent cells. [25]. Several studies have demonstrated that AMPs affect the phagocytic capacity of macrophages and thus regulate immunity [27,28,29]. Wang et al. reported that sublancin, a glucosylated AMP isolated from *Bacillus subtilis* 168, significantly enhanced the phagocytic activity of macrophages in vivo and in vitro [24]. In addition, Wan et al. demonstrated that LL-37 is a 37 amino acid cationic AMP that increases phagocytosis by human macrophages [28]. Furthermore, it is known that retrocyclins, humanized analogs of the theta-defensin AMP, also enhance phagocytosis by macrophages [29]. Consistent with the above notions, our results suggested that CSP32 stimulated phagocytosis while inducing the appearance of a typical M1 polarized macrophage phenotype that included spindle-like morphology with elongated filopodia (Figure 1).

It is known that activated macrophages phagocytose the inflammasome complex to further induce inflammation [26]. M1 macrophages are stimulated by Toll-like receptor (TLR) ligands and/or Th1 cytokines, including LPS, interferon-γ (IFN-γ), and TNF-α [5]. These macrophages produce and release high levels of pro-inflammatory cytokines (e.g., IL-1α, IL-1β, IL-6, IL-12, IL-23, TNF-α, and COX-2), chemokines (e.g., CXCL1-3, CXCL-5, and CXCL8-10), NO, and reactive oxygen species [5]. Interestingly, AMP can encourage both innate immunity by interacting with TLRs and acquired immunity by acting as adjuvants [30,31]. Furthermore, AMPs regulate the expression of chemokine and chemokine receptors in macrophages and produce the secretion of cytokines or chemokines by different immune cells [32]. To investigate whether CSP32 modulates the production and secretion of M1 macrophage-stimulated proinflammatory mediators, we performed gene expression microarray, RT-PCR, ELISA, and western blot analyses. Our results showed that CSP32 significantly enhanced the expression and secretion of markers for M1 macrophages, including pro-inflammatory cytokines and chemokines genes (Figure 2 and Figure 3). In particular, the secretion levels of pro-inflammatory mediators, such as NO, PGE_2_, TNF-α, IL-1β, and MCP-1, were markedly elevated by CSP32, and this finding is consistent with the expression of mRNA and protein. On the contrary, CSP32 downregulated the expression of anti-inflammatory genes and apoptosis regulatory genes (Figure 2). In fact, apoptotic cells are divers of M2-like activation of macrophage and suppress anti-tumor activity of M1 macrophages. Apoptosis-mediated M1 suppression could lead to repolarization from M1 to M2-like tumor supportive cells [33]. In respect, our finding suggested that CSP32 suppressed M2 polarization which is characterized by anti-inflammation and apoptosis.

The interrelated actions of various inflammatory mediators, signaling molecules, and transcriptional factors are involved in MP regulation [34]. It is well known that the canonical IFN-recognition factor (IRF)/STAT signaling pathway is critical in MP regulation. INF-γ triggers the JAK/STAT (e.g., STAT 1 and STAT3) signaling pathway, subsequently promoting target inflammatory genes, such as IL-12 and NO [35,36]. Moreover, activated STAT signaling is involved in TLR4/NF-κB-mediated transcription of target genes, and these signals culminate in the production of M1-associated inflammatory mediators [7]. In this regard, numerous studies have established that the activation of the NF-κB signaling pathway accounts for M1 macrophages that subsequently mediate inflammation [37,38]. Li et al. demonstrated that cathelicidin-related AMPs are important effectors of innate immunity and induce the recruitment of inflammatory cells through the NF-κB p65/RelA signaling pathway [39]. More recently, it was reported that glucosylated AMP modulates immunity by inducing proinflammatory mediators, which are partly dependent on the TLR4/MAPK/NF-κB signaling pathway [27]. Herein, our findings showed that CSP32 markedly upregulated the gene expression of IRFs, JAKs, STATs, and NF-κB according to the results of the nCounter microarray (Figure 2). In addition, our results clearly demonstrated that CSP32 induced NF-κB activation through phosphorylation of its upstream MAPK signaling molecules (Figure 4). Taken together, these data indicated that CSP32-stimulated inflammatory mediators can be partially mediated by the JAK/STAT signaling pathway and mainly mediated by the MAPK/NF-κB signaling pathway during M1 macrophage polarization. However, the upstream factors interacting with the MAPK/NF-κB signaling pathways, which culminate in CSP32-mediated M1 macrophages, are poorly understood. Thus, we focused on the change in intracellular Ca^2+^ during the process of CSP-induced M1 polarization.

Intracellular Ca^2+^ is an important divalent cation that controls many cellular functions, such as cell plasticity, development, cell proliferation, and differentiation [40]. It is well known that the PLC/inositol 1,4,5-trisphosphate (IP_3_)/diacylglycerol (DAG) signaling pathway activates PKCs and Ca^2+^ signals [41]. Interestingly, increased levels of intracellular Ca^2+^ promote immune functions, and decreased intracellular Ca^2+^ suppresses phagocytosis and the production of inflammatory mediators in the J774 macrophage cell line [42,43]. As shown in Figure 5A,B, CSP32 markedly increased both the population and the expression of Ca^2+^-positive macrophages. In addition, our findings showed that CSP32 upregulated the PLCγ/PKCε signaling pathway, which is well known to be upstream of calcium signaling (Figure 5C). Furthermore, inhibition of intracellular Ca^2+^ by BAPTA-AM significantly suppressed CSP32-mediated phagocytosis, inflammatory mediator production, and NF-κB activation (Figure 6). Cytosolic Ca^2+^ changes occur mainly due to the release of Ca^2+^ from endoplasmic reticulum stores (ER) and due to influx through the plasma membrane Ca^2+^, which represent store-operated Ca^2+^ entry (SOCE) [44]. Accumulating studies have focused on the role of Ca^2+^ release-activated Ca^2+^ (CRAC) channels in immune cell function, but their role in the macrophage responses to regulate immunity is limited [45]. In 2018, Chauhan et al. demonstrated that transient receptor potential canonical channel (TRPC) 1-mediated Ca^2+^ influx is essential for the induction of MP to the M1 inflammatory phenotype, while ORAI1 (a gene encoding CRAC)-mediated basal Ca^2+^ influx regulated naive macrophages [46]. Although we show that CSP32 enhances Ca^2+^ influx and ultimately induces M1 polarization via the NF-κB signaling pathway, the study design does not address which calcium channels contribute to the CSP32 stimulation-induced Ca^2+^ influx. Therefore, further studies are required to investigate the release and influx of Ca^2+^ from the ER and plasma membrane and to assess the expression of calcium channels in the development of CSP32-stimulated M1 inflammatory macrophages.

## 5. Conclusions

**Figure 7:**
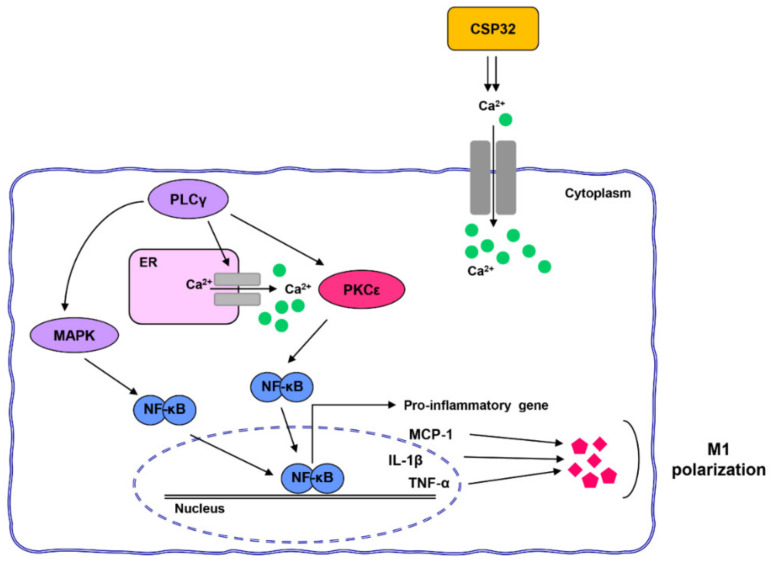
CSP32 stimulates M1 polarization via the calcium-dependent NF-κB signaling pathway.

Taken together, CSP32 enhanced phagocytosis and inflammation via the NF-κB signaling pathway and led to M1 macrophage polarization. Importantly, CSP32-stimulated M1 polarization is dependent on the PLCγ/PKCε/calcium signaling pathway (Figure 7). Our findings suggested that CSP32 induced M1 macrophage polarization via the intracellular Ca^2+^ influx and may result in enhanced immune capacities. Although further studies are needed to identify the calcium channels in CSP32-stimulated macrophages, it is worth noting that the immune-enhancing effect of CSP32 is dependent on the calcium signaling pathway. Furthermore, further studies are needed to accurately establish whether CSP32 has an immune-enhancing effect in human-derived M1 and M2 polarized cell lines, such as GM-CSF and M-CSF cells.

## Supplementary Materials

The following are available online at https://www.mdpi.com/2072-6643/12/6/1603/s1, Table S1: Primary and secondary antibodies used for immunoblotting. Figure S1: Histogram shows the log2-fold change of each genes in expression between untreated cells and 88.0 μM CSP32-treated cells. Figure S2: CPS32-mediated M1 macrophage polarization regulates by PLC signaling pathway.

## References

[1] Tauber A.I. (2003). Metchnikoff and the phagocytosis theory. Nat. Rev. Mol. Cell Biol..

[2] Parihar A., Eubank T.D., Doseff A.I. (2010). Monocytes and macrophages regulate immunity through dynamic networks of survival and cell death. J. Innate. Immun..

[3] Murray P.J., Wynn T.A. (2011). Protective and pathogenic functions of macrophage subsets. Nat. Rev. Immunol..

[4] Mosser D.M., Edwards J.P. (2008). Exploring the full spectrum of macrophage activation. Nat. Rev. Immunol..

[5] Shapouri-Moghaddam A., Mohammadian S., Vazini H., Taghadosi M., Esmaeili S.A., Mardani F., Seifi B., Mohammadi A., Afshari J.T., Sahebkar A. (2018). Macrophage plasticity, polarization, and function in health and disease. J. Cell Physiol..

[6] Biswas S.K., Mantovani A. (2010). Macrophage plasticity and interaction with lymphocyte subsets: Cancer as a paradigm. Nat. Immunol..

[7] Wu X.Q., Dai Y., Yang Y., Huang C., Meng X.M., Wu B.M., Li J. (2016). Emerging role of microRNAs in regulating macrophage activation and polarization in immune response and inflammation. Immunology.

[8] Lei J., Sun L., Huang S., Zhu C., Li P., He J., Mackey V., Coy D.H., He Q. (2019). The antimicrobial peptides and their potential clinical applications. Am. J. Transl. Res..

[9] Bahar A.A., Ren D. (2013). Antimicrobial peptides. Pharmaceuticals.

[10] Ruiu L. (2013). Brevibacillus laterosporus, a pathogen of invertebrates and a broad-spectrum antimicrobial species. Insects.

[11] Claus D., Berkeley R.C.W., Sneath P.H.A., Mair N.S., Sharpe M.E., Holt J.G. (1986). The Genus Bacillus. Bergey’s Manual of Systematic Bacteriology.

[12] Abriouel H., Franz C.M., Ben Omar N., Gálvez A. (2011). Diversity and applications of Bacillus bacteriocins. FEMS Microbiol. Rev..

[13] Stein T. (2005). Bacillus subtilis antibiotics: Structures, syntheses and specific functions. Mol. Microbiol..

[14] Tamang J.P., Watanabe K., Holzapfel W.H. (2016). Review: Diversity of microorganisms in global fermented foods and beverages. Front Microbiol..

[15] Choi Y.H., Cho S.S., Simkhada J.R., Rahman M.S., Choi Y.S., Kim C.S., Yoo J.C. (2017). A novel multifunctional peptide oligomer of bacitracin with possible bioindustrial and therapeutic applications from a Korean food-source Bacillus strain. PLoS ONE.

[16] Kwon D.H., Cha H.J., Lee H., Hong S.H., Park C., Park S.H., Kim G.Y., Kim S., Kim H.S., Hwang H.J. (2019). Effect of glutathione against oxidative stress-induced cytotoxicity in RAW 264.7 macrophages through activating the nuclear factor erythroid 2-related factor-2/heme oxygenase-1 pathway. Antioxidants.

[17] Guo S., Al-Sadi R., Said H.M., Ma T.Y. (2013). Lipopolysaccharide causes an increase in intestinal tight junction permeability in vitro and in vivo by inducing enterocyte membrane expression and localization of TLR-4 and CD14. Am. J. Pathol..

[18] Schwartz E.A., Zhang W.Y., Karnik S.K., Borwege S., Anand V.R., Laine P.S., Su Y., Reaven P.D. (2010). Nutrient modification of the innate immune response: A novel mechanism by which saturated fatty acids greatly amplify monocyte inflammation. Arterioscler. Thromb. Vasc. Biol..

[19] Lee M.H., Hong S.H., Park C., Han M.H., Kim S.O., Hong S.H., Kim G.Y., Choi Y.H. (2017). Anti-inflammatory effects of Daehwangmokdantang, a traditional herbal formulation, in lipopolysaccharide-stimulated RAW 264.7 macrophages. Exp. Ther. Med..

[20] Kwon D.H., Lee H., Park C., Hong S.H., Hong S.H., Kim G.Y., Cha H.J., Kim S., Kim H.S., Hwang H.J. (2019). Glutathione induced immune-stimulatory activity by promoting M1-like macrophages polarization via potential ROS scavenging capacity. Antioxidants.

[21] Kulkarni M.M. (2011). Digital multiplexed gene expression analysis using the NanoString nCounter system. Curr. Protoc. Mol. Biol..

[22] Park C., Cha H.J., Lee H., Hwang-Bo H., Ji S.Y., Kim M.Y., Hong S.H., Jeong J.W., Han M.H., Choi S.H. (2019). Induction of G2/M cell cycle arrest and apoptosis by genistein in human bladder cancer T24 cells through inhibition of the ROS-dependent PI3k/Akt signal transduction pathway. Antioxidants.

[23] Gu Z.T., Li L., Wu F., Zhao P., Yang H., Liu Y.S., Geng Y., Zhao M., Su L. (2015). Heat stress induced apoptosis is triggered by transcription-independent p53, Ca^2+^ dyshomeostasis and the subsequent Bax mitochondrial translocation. Sci. Rep..

[24] Mantovani B., Rabinovitch M., Nussenzweig V. (1972). Phagocytosis of immune complexes by macrophages. Different roles of the macrophage receptor sites for complement (C3) and for immunoglobulin (IgG). J. Exp. Med..

[25] Nakamura Y., Fukami K. (2017). Regulation and physiological functions of mammalian phospholipase C. J. Biochem..

[26] Aderem A., Underhill D.M. (1999). Mechanisms of phagocytosis in macrophages. Annu. Rev. Immunol..

[27] Hirayama D., Iida T., Nakase H. (2017). The Phagocytic function of macrophage-enforcing innate immunity and tissue homeostasis. Int. J. Mol. Sci..

[28] Wang S., Ye Q., Wang K., Zeng X., Huang S., Yu H., Ge Q., Qi D., Qiao S. (2019). Enhancement of macrophage function by the antimicrobial peptide sublancin protects mice from methicillin-resistant *Staphylococcus aureus*. J. Immunol. Res..

[29] Wan M., van der Does A.M., Tang X., Lindbom L., Agerberth B., Haeggström J.Z. (2014). Antimicrobial peptide LL-37 promotes bacterial phagocytosis by human macrophages. J. Leukoc. Biol..

[30] Welkos S., Cote C.K., Hahn U., Shastak O., Jedermann J., Bozue J., Jung G., Ruchala P., Pratikhya P., Tang T. (2011). Humanized theta-defensins (retrocyclins) enhance macrophage performance and protect mice from experimental anthrax infections. Antimicrob. Agents. Chemother..

[31] Biragyn A., Ruffini P.A., Leifer C.A., Klyushnenkova E., Shakhov A., Chertov O., Shirakawa A.K., Farber J.M., Segal D.M., Oppenheim J.J. (2002). Toll-like receptor 4-dependent activation of dendritic cells by beta-defensin 2. Science.

[32] Lillard J.W., Boyaka P.N., Chertov O., Oppenheim J.J., McGhee J.R. (1999). Mechanisms for induction of acquired host immunity by neutrophil peptide defensins. Proc. Natl. Acad. Sci. USA.

[33] Weigert A., Tzieply N., von Knethen A., Johann A.M., Schmidt H., Geisslinger G., Brüne B. (2007). Tumor cell apoptosis polarizes macrophages role of sphingosine-1-phosphate. Mol. Biol. Cell.

[34] Scott M.G., Davidson D.J., Gold M.R., Bowdish D., Hancock R.E. (2002). The human antimicrobial peptide LL-37 is a multifunctional modulator of innate immune responses. J. Immunol..

[35] Kong L.N., Lin X., Huang C., Ma T.T., Meng X.M., Hu C.J., Wang Q.Q., Liu Y.H., Shi Q.P., Li J. (2019). Hesperetin derivative-12 (HDND-12) regulates macrophage polarization by modulating JAK2/STAT3 signaling pathway. Chin. J. Nat. Med..

[36] Wang S., Cao M., Xu S., Shi J., Mao X., Yao X., Liu C. (2020). Luteolin alters macrophage polarization to inhibit inflammation. Inflammation.

[37] Wang N., Liang H., Zen K. (2014). Molecular mechanisms that influence the macrophage m1-m2 polarization balance. Front. Immunol..

[38] Du L., Li J., Zhang X., Wang L., Zhang W., Yang M., Hou C. (2019). Pomegranate peel polyphenols inhibits inflammation in LPS-induced RAW264.7 macrophages via the suppression of TLR4/NF-κB pathway activation. Food Nutr. Res..

[39] Liu C.P., Zhang X., Tan Q.L., Xu W.X., Zhou C.Y., Luo M., Li X., Huang R.Y., Zeng X. (2017). NF-κB pathways are involved in M1 polarization of RAW 264.7 macrophage by polyporus polysaccharide in the tumor microenvironment. PLoS ONE.

[40] Li D., Beisswenger C., Herr C., Schmid R.M., Gallo R.L., Han G., Zakharkina T., Bals R. (2014). Expression of the antimicrobial peptide cathelicidin in myeloid cells is required for lung tumor growth. Oncogene.

[41] Feske S., Wulff H., Skolnik E.Y. (2015). Ion channels in innate and adaptive immunity. Annu. Rev. Immunol..

[42] Chen B.C., Chou C.F., Lin W.W. (1998). Pyrimidinoceptor-mediated potentiation of inducible nitric-oxide synthase induction in J774 macrophages. Role of intracellular calcium. J. Biol. Chem..

[43] Watanabe N., Suzuki J., Kobayashi Y. (1996). Role of calcium in tumor necrosis factor-alpha production by activated macrophages. J. Biochem..

[44] Pani B., Bollimuntha S., Singh B.B. (2012). The TR (i)P to Ca^2+^ signaling just got STIMy: An update on STIM1 activated TRPC channels. Front. Biosci..

[45] Feske S. (2010). CRAC channelopathies. Pflugers Arch..

[46] Chauhan A., Sun Y., Sukumaran P., Quenum Zangbede F.O., Jondle C.N., Sharma A., Evans D.L., Chauhan P., Szlabick R.E., Aaland M.O. (2018). M1 macrophage polarization is dependent on TRPC1-mediated calcium entry. iScience.

