# Correction: Ji et al. A Novel Peptide Oligomer of Bacitracin Induces M1 Macrophage Polarization by Facilitating Ca^2+^ Influx. *Nutrients* 2020, *12*, 1603

**DOI:** 10.3390/nu17233723

**Published:** 2025-11-27

**Authors:** Seon Yeong Ji, Hyesook Lee, Hyun Hwangbo, Su-Hyun Hong, Hee-Jae Cha, Cheol Park, Do-Hyung Kim, Gi-Young Kim, Suhkmann Kim, Heui-Soo Kim, Jin Cheol Yoo, Yung Hyun Choi

**Affiliations:** 1 Anti-Aging Research Center, Dong-eui University, Busan 47340, Republic of Korea; 14602@deu.ac.kr (S.Y.J.); lhyes0219@pusan.ac.kr (H.L.); hbhyun2003@naver.com (H.H.); hongsh@deu.ac.kr (S.-H.H.); 2 Department of Biochemistry, College of Korean Medicine, Dong-eui University, Busan 47227, Republic of Korea; 3 Department of Parasitology and Genetics, College of Medicine, Kosin University, Busan 49104, Republic of Korea; hcha@kosin.ac.kr; 4 Department of Molecular Biology, College of Natural Sciences, Dong-eui University, Busan 47340, Republic of Korea; parkch@deu.ac.kr; 5 Department of Aquatic Life Medicine, College of Fisheries Science, Pukyong National University, Busan 48513, Republic of Korea; dhkim@pknu.ac.kr; 6 Department of Marine Life Sciences, School of Marine Biomedical Sciences, Jeju National University, Jeju 63243, Republic of Korea; immunkim@jejunu.ac.kr; 7 Department of Chemistry, College of Natural Sciences, Pusan National University, Busan 46241, Republic of Korea; suhkmann@pusan.ac.kr; 8 Department of Biological Sciences, College of Natural Sciences, Pusan National University, Busan 46241, Republic of Korea; khs307@pusan.ac.kr; 9 Department of Pharmacy, College of Pharmacy, Chosun University, Gwangju 61452, Republic of Korea; jcyu@chosun.ac.kr

In the original publication [1], there was a mistake in Figures 1E and 3G. This data error occurred due to an unforeseen mistake during the data organization process. The correct Figure 1 and Figure 3 appear below. The authors state that the scientific conclusions are unaffected. This correction was approved by the Academic Editor. The original publication has also been updated.

**Figure 1:**
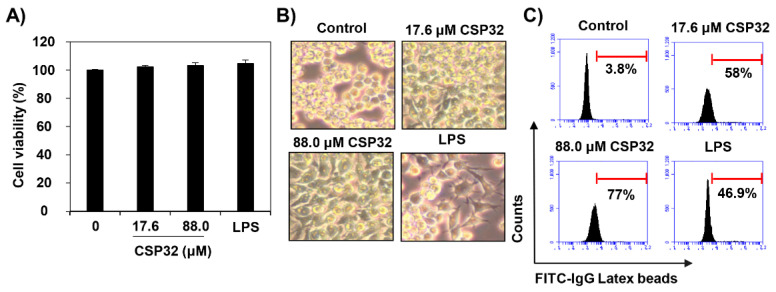

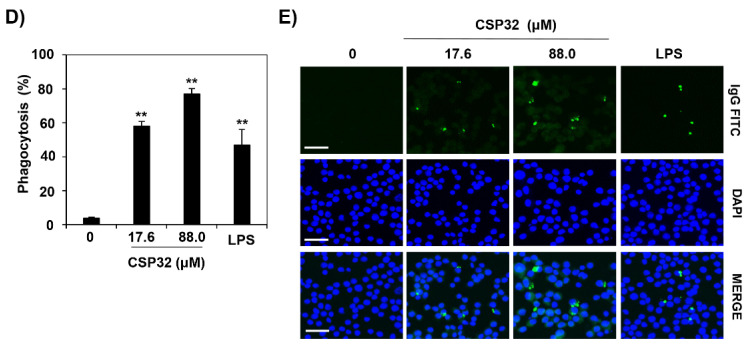
CSP32 induced morphological changes and phagocytosis of macrophages. Cells were treated with the indicated concentrations of CSP32 and LPS for 24 h. (**A**) Cell viability was assessed by MTT assay. (**B**) Representative microscopy images of morphological changes. (**C**,**D**) Representative flow cytometric histogram and quantitative analysis of the phagocytosis capacity using fluorescent FITC-IgG latex beads. Data are expressed as the mean ± SD (*n* = 4). ** *p* <0.01 compared with the control. (**E**) The phagocytic cells were visualized by fluorescence microscopy. The nuclei were stained with DAPI. Scale bar: 200 μm.

**Figure 3:**
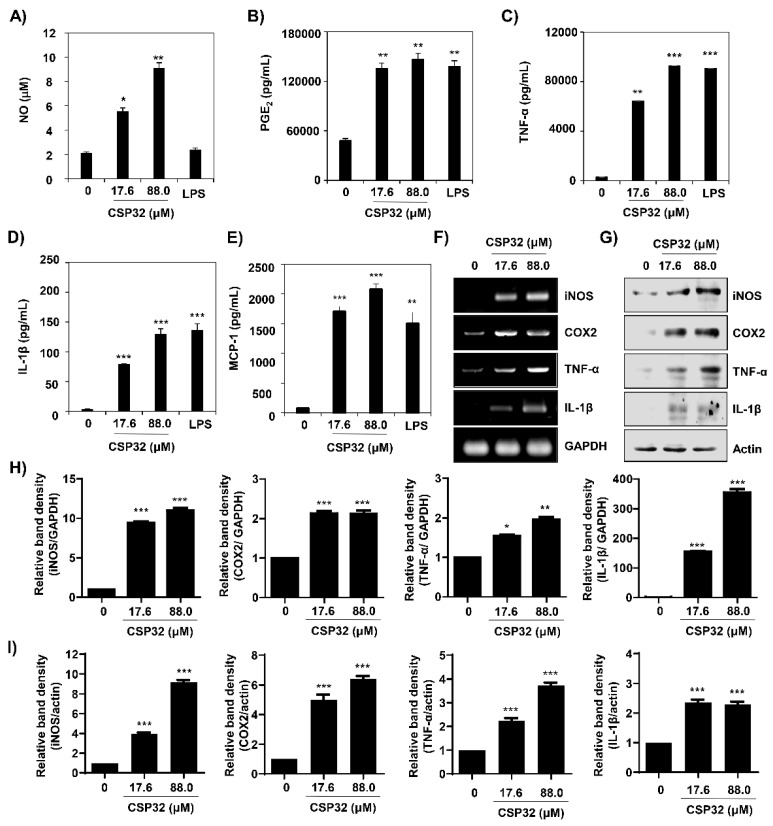
CSP32 increased the levels of markers for M1 macrophages. Cells were treated with the indicated concentrations of CSP32 and LPS for 24 h. (**A**) The amount of NO in the cell supernatant was measured using Griess reagents. The levels of PGE_2_ (**B**), TNF-α (**C**), IL-1β (**D**), and MCP-1 (**E**) in the culture supernatants were measured by ELISA kits. Data are expressed as the mean ± SD (*n* = 4). * *p* < 0.05, ** *p* < 0.01, and *** *p* < 0.001 compared with the control. mRNA (**F**) and protein (**G**) expression of markers of M1 macrophages, including iNOS, COX-2, TNF-α, and IL-1β. GAPDH and β-actin were used as internal controls for RT-PCR and Western blotting. Quantitative analysis of mRNA (**H**) and protein (**I**) expression. The expression of each protein was indicated as a fold change relative to the control. Data are expressed as the mean ± SD (*n* = 3). * *p* < 0.05, ** *p* < 0.01, and *** *p* < 0.001 compared with the control.
